# Interaction of Fluorescently Labeled Cadherin G Protein-coupled Receptor with the Cry1Ab Toxin of Bacillus thuringiensis

**DOI:** 10.4172/jpb.1000474

**Published:** 2018-05-14

**Authors:** Li Liu, Stefanie Boyd, Mehraban Kavoussi, Lee A Bulla, Duane D. Winkler

**Affiliations:** Department of Biological Sciences, University of Texas at Dallas, Richardson, Texas 75080, United States of America

**Keywords:** GPCR, Cadherin, Cry1Ab, Fluorescent labeling, C-maleimide

## Abstract

The Cry1Ab toxin produced by *Bacillus thuringiensis* binds to a conserved structural motif in the 12^th^ ectodomain module (EC12) of BT-R_1_, a cadherin G protein-coupled receptor (GPCR) contained in the membrane of midgut epithelial cells of the tobacco hornworm *Manduca sexta*. Toxin binding transmits a signal into the cells and turns on a multi-step signal transduction pathway, culminating in cell death. Using chromatographically purified Cry1Ab and EC12 proteins, we demonstrated the direct formation of a stable complex between these two proteins in solution and visualized it on a native polyacrylamide gel. Moreover, we generated a fluorescent EC12 probe by converting the 36^th^ residue to cysteine to enable maleimide-mediated conjugation of Alexa-488 fluorescent dye to EC12 by site-directed mutagenesis. In addition, we changed the 44^th^ residue of EC12 to tryptophan, which greatly improved accuracy of protein quantification and traceability. Using the fluorescently labeled EC12 probe for direct and competitive binding assays, we were able to determine binding specificity in solution. These accomplishments will facilitate identification and characterization of the interface sequences for both the Cry1Ab toxin and BT-R_1_.

## Introduction

*Bacillus thuringiensis* (Bt) is a group of spore-forming Grampositive bacteria that produce entomocidal parasporal crystalline proteins during the sporulation phase of their life cycle [1]. The proteins (Cry toxins) exert their insecticidal action on a variety of insects including moths, beetles and mosquitoes, many of which are economically important to agriculture and public health. The lethal action of Cry toxins is initiated by binding of the toxins to specific receptors in the midgut epithelium of susceptible insect larvae. These receptors, represented by BT-R_1_ of the moth *Manduca sexta* (tobacco hornworm) [2,3], constitute a family of homologous cadherin G protein-coupled receptors (GPCRs) that are essential to the binding and activity of the various Cry toxins [1,3–7]. BT-R_1_ is composed of four domains: Ectodomain (EC), Membrane-Proximal Extracellular Domain (MPED), Transmembrane Domain (TM) and Cytoplasmic Domain (CYTO) [4]. The EC contains 12 ectodomain modules (EC1–EC12), each of which is composed of β-barrel cadherin repeats and are connected one to another by interdomain linkers. Previously, we demonstrated that cadherin GPCR signaling is ligand (Cry toxin)-induced in the tobacco hornworm and is a general phenomenon in other insect systems as well [1]. The primary focus in this report is on the 210-kDa cadherin receptor BT-R_1_, which is present in midgut epithelial cells of tobacco hornworm larvae [4]. The 65-kDa Cry1Ab toxin produced by Bt binds to a conserved structural motif within the 12^th^ ectodomain (EC12) of BT-R_1_ [8]. This incident is essential for toxicity of Cry1Ab to the insect. Binding of the toxin to BT-R_1_ triggers a signaling event, which stimulates its coupled heterotrimeric G protein. The G protein, in turn, activates an effector enzyme, adenylyl cyclase, that catalyzes a dramatic increase in the intracellular second messenger cAMP [9]. cAMP activates protein kinase A, bringing about cytological rearrangement and ion fluxing as well as other significant changes such as enhanced exocytosis of BT-R_1_ from intracellular membrane vesicles to the cell surface [10]. Cry1Ab-induced enrichment of BT-R_1_ on the cell surface amplifies the signaling and sets the stage for additional toxin binding, leading to enhanced cell death. Cry1Ab toxin binding to BT-R _1_ is specific and selective in either cultured insect cells or whole insects [1,3,4,11,12]. Indeed, EC12, the proximal cadherin repeat of the BT-R_1_ ectodomain, completely inhibits Cry1Ab toxin action in insects when it is pre-incubated with the toxin at a 1:1 molar ratio [4]. Moreover, BT-R _1_ toxicity of High Five cells transfected with BT-R_1_ cDNA is inhibited maximally when soluble EC12 is present at a 1:1 ratio with Cry1Ab [8]. Based on our cytological and biochemical investigations, it is evident that univalent binding of the Cry1Ab toxin affects the structural and functional properties of BT-R_1_ to bring about cellular damage and total loss of function in midgut epithelial cells of tobacco hornworm larvae exposed to the toxin. We are interested particularly in determining the amino acid residues that define both the toxin-binding region within the EC12 module of BT-R_1_ and the receptor-binding region within the Cry1Ab toxin. Binding of the two proteins depends on the N- and C-terminal ends of EC12, which fashion the boundaries of the module and define its structural motif. Sequences in the two ends are highly conserved among homologous cadherin GPCRs in different moth species. Excluding the two ends abolishes binding of EC12 to Cry1Ab and Cry1Ab toxicity [8]. These two ends undoubtedly mediate Cry1Ab interaction with EC12 and, most certainly, promote Cry1Ab toxicity. To further examine those features that define the binding of the toxin to BT-R_1_ in the context of specificity and affinity, we chromatographically purified both the Cry1Ab toxin and the toxin-binding region, EC12, of BT-R_1_ and examined the direct interaction of the two molecules in solution. Additionally, we constructed a fluorescent EC12 probe for determining the specificity and affinity of EC12 binding to Cry1Ab by converting residue 36 (serine) in EC12 to cysteine. This change permitted C-maleimide mediated conjugation of Alexa Fluor® 488 to the free thiol group on EC12. We also introduced a tyrosine to tryptophan change at residue 44 of EC12 for better quantification of the protein and easier tracking during the purification process. The fluorescence signal was exceptionally strong and uniform and, notably, did not interfere with binding of the toxin to EC12. Hence, we relied on the high-sensitivity probe (EC12_36C/44W_) to examine various binding properties of Cry1Ab and EC12. By monitoring the interaction of fluorescently labeled EC12 and Cry1Ab toxin in a non-denaturing polyacrylamide gel system, we verified formation of a highly stable, soluble complex that most likely typifies such structures formed in a variety of related moth species.

## Materials and Methods

### Cloning, site-directed mutagenesis and expression of the BT-R_1_ toxin-binding region (EC12) proteins

Cloning and characterization of BT-R_1_ (GenBank accession no. AF310073) was done previously in our laboratory [2–4]. The DNA fragment encoding wild-type EC12 (EC12_WT_; amino acid residues 1348–1459) was generated by PCR amplification. The fragment was cloned directionally between the *SalI* and *HindIII* sites of the pHAT4 6xHis tag fusion plasmid vector, which encodes for an N-terminal 6xHis tag followed by a specific recognition and cleavage site for a protease encoded by tobacco etch virus (TEV protease). pHAT4 conveys ampicillin resistance and utilizes a T7 RNA polymerase promoter and terminator for expression [13]. Mutations were introduced into EC12_WT_ using the Phusion Site-Directed Mutagenesis Kit (Thermo Fisher Scientific) and changes to amino acid residues 36 (serine changed to cysteine) and 44 (tyrosine changed to tryptophan) of EC12 were generated. EC12_44W_ was constructed to facilitate detection of protein during purification using a stain-free method (see below). The EC12_36C/44W_ was constructed specifically to enable labeling with Alexa-488 fluorescent dye (see below). Sequences of all recombinant plasmid constructs were confirmed by Sanger sequencing. Confirmed EC12 constructs were transformed into BL21 (DE3) pLysS *Escherichia coli* (Promega) for protein expression. Selected recombinant bacterial colonies were grown overnight at 37°C in 2X YT medium containing ampicillin (100 μg/mL) to obtain a starter culture. The starter culture was inoculated into 10X volume of 2X YT medium next day and grown with shaking (250 rpm) at 37°C to OD_600 nm_ = 0.8. Expression of the recombinant proteins was induced by the addition of 1 M isopropyl β-thiogalactoside (IPTG) to a final concentration of 500 μM for 4 h at 37°C. Cells were harvested by centrifugation at 8000 rpm for 15 minutes at 4°C and re-suspended in buffer containing 20 mM Tris pH 8.0, 300 mM NaCl, 2 mM dithiothreitol (DTT) and 1 mM phenylmethanesulfonyl fluoride (PMSF), followed by sonication.

### Purification of recombinant proteins

Soluble proteins were collected after centrifugation at 18,000 rpm for 30 min at 4°C and filtered. Protein purification was carried out with a HisTrap HP affinity column (GE Healthcare Life Sciences) equilibrated with cell re-suspension buffer on an ÄKTA pure protein purification system (GE Healthcare Life Sciences). Elution of proteins was carried out in a gradient of cell re-suspension buffer and 20 mM Tris buffer pH 8.0 supplemented with 300 mM NaCl, 2 mM DTT and 1 M imidazole. Fractions containing EC12 were pooled and separated on a HiTrap Q HP column (GE Healthcare Life Sciences) equilibrated with 20 mM Tris pH 8.0 plus 2 mM DTT and eluted in a gradient of 20 mM Tris pH 8.0 supplemented with 1 M NaCl and 2 mM DTT. At the last step, the 6xHis tag on the recombinant protein was removed by a protease encoded by the TEV. HisTrap chromatography was used to separate untagged recombinant proteins. The purified protein was concentrated and frozen for future use.

### Purification of Cry 1Ab toxin

*Bacillus thuringiensis* subsp*. berliner* was cultured in a MnCl_2_-containing trypticase broth (pH 7.0) with constant shaking (200 rpm) at 30°C for 70 h. Purification of the Cry1Ab toxin was accomplished as previously described with slight modification [8]. Briefly, sporulated cells containing parasporal crystals (composed of 130-kDa protoxin) were harvested by centrifugation at 7,000 rpm for 10 min at 4°C. The pelleted cells were re-suspended in a 50 mM Na_2_CO_3_ buffer pH 10.1 containing 2 mM DTT and shaken (200 rpm) at room temperature for 2 h to facilitate crystal protoxin dissolution. Soluble Cry1Ab protoxin was recovered by centrifuging at 16,000 rpm for 10 min at 4°C and dialyzed overnight at 4°C against 50 mM Tris (pH 8.7) supplemented with 50 mM NaCl and 2 mM DTT. Treatment of the recovered soluble protoxin with trypsin (Cat# 27250-018, Life Technologies) at 37°C for 2 h rendered soluble, activated 65-kDa Cry1Ab toxin, which was purified with a HiTrap Q HP chromatography column on an ÄKTA pure system.

### Stain-free imaging, immunoligand blot analysis and fluorescence-based detection of toxin binding to EC12

Protein samples were heated at 95°C for 5 min in SDS-PAGE sample buffer and subjected to SDS-PAGE (Mini-Protean Gel Electrophoresis System, Bio-Rad) using gels containing 0.5% 2,2,2-trichloroethanol (TCE, Sigma-Aldrich). Crosslinking (covalent binding) of the tryptophan residues in the proteins to TCE was activated by ultraviolet light [14]. The resulting tryptophan adducts emitted fluorescence upon excitation by further irradiation, which was captured by a ChemiDoc MP Imaging System (Bio-Rad). This stain-free procedure minimizes any permanent modifications of the proteins and allows use of the same gel for western blot analysis. The proteins resolved on the stain-free gels were electro-transferred directly to nitrocellulose membranes using the Trans-Blot Turbo Transfer System (Bio-Rad). Membranes were blocked in 5% dry-milk containing Tris-buffered saline supplemented with 0.5% Tween-20 (TBS-T) for 30 min. To detect His-tagged proteins during purification, membranes were incubated in 1:5,000 diluted monoclonal 6x-His Epitope Tag Antibody (MA1-21315, Invitrogen) for 2 h at room temperature followed by two washes with TBS-T buffer. Membranes were further incubated with horseradish peroxidase-conjugated goat anti-mouse antibody (1:10,000; ThermoFisher Scientific, Cat# 31432) for 1 h followed by four washes with TBS-T buffer. Protein bands were developed using Amersham ECL Western Blotting Prime Detection Reagent and visualized in a ChemiDoc MP Imaging System (Bio-Rad).

### Maleimide-mediated conjugation of Alexa-488 to EC12_36C/44W_

Alexa-488 dye (Thermo Fisher Scientific) was stably conjugated to the thiol groups of cysteine of the purified EC12_36C/44W_ with a maleimide reagent (Life Technologies) according to the manufacturer’s instructions. This reaction was carried out at 4°C with mixing of Alexa-488 and EC12_36C/44W_ peptide at an equimolar ratio overnight in buffer containing 20 mM Tris (pH 7.5), 150 mM NaCl and 1 mM tris (2-carboxyethyl) phosphine. Both dithiothreitol and β-mercaptoethanol should not be used in the reaction because each of them contains free thiols. Labeled protein was separated from free dye molecules by size exclusion chromatography (Superdex 200). The fluorescently labeled EC12_36C/44W_ (EC12 probe or probe) was aliquoted and frozen in −80°C for in-solution and competitive binding assays.

### In-solution binding of Cry1Ab toxin to EC12 and competition binding assays

Binding of native Cry1Ab to EC12_WT_ or non-fluorescently labeled EC12_36C/44W_ or EC12_44W_ was conducted in a solution containing 20 mM Tris (pH 8.0) supplemented with 200 mM NaCl, 1 mM DTT and 5% glycerol for 20 min at room temperature. Reaction volume was 10 μl. Samples were resolved on a native PAGE gel and visualized by silver staining. Binding reactions of fluorescently labeled EC12_36C/44W_ with Cry1Ab were conducted under the same condition to accurately determine the Cry1Ab/EC12 complex. Formation of the Cry1Ab/EC12_36C/44W_ complex and disappearance of the EC12_36C/44W_ were monitored by using a Typhoon FLA 9500 (GE Healthcare Life Sciences). In addition, competitive binding experiments were done to determine the specificity of Cry1Ab binding to EC12 alone with the fluorescently labeled EC12_36C/44W_ probe. Incubation of Cry1Ab with unlabeled mutants (EC12_36C/44W_ or EC12_44W_) and EC12_WT_ molecules at various concentrations was carried out at room temperature for 15 min. Fluorescently labeled EC12_36C/44W_ then was added to each reaction and incubated for an additional 20 min upon which they were examined to discern competition on a 6% native PAGE gel.

## Results

**Figure 1:**
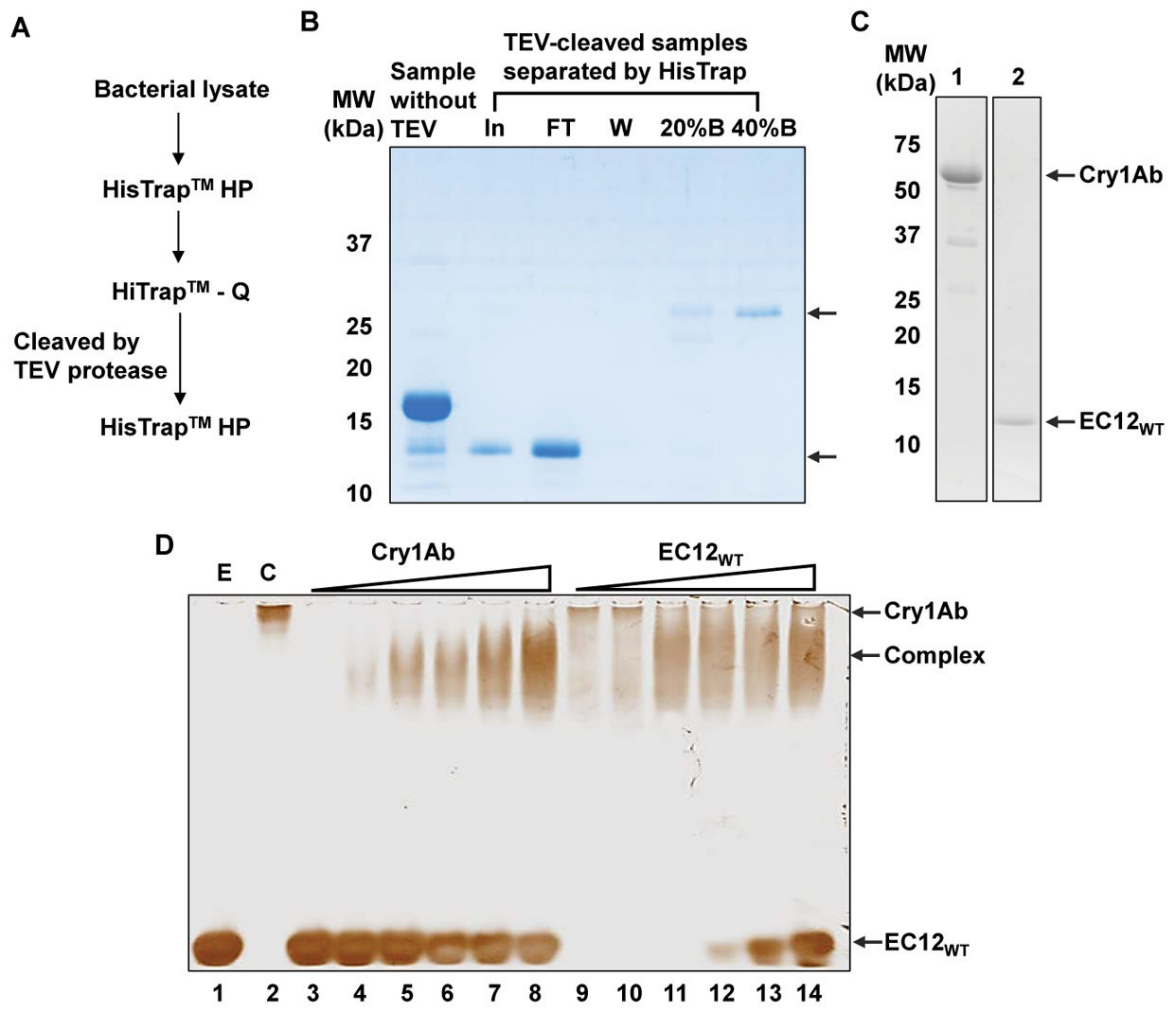
Binding of purified EC12 to native Cry1Ab toxin in solution. (A) Schematic showing the chromatographic purification steps applied to purify the recombinant wild-type EC12 (EC12_WT_) protein. Details of the purification procedure are described in “Materials and Methods”. Throughout the purification procedure, presence of the 6xHis tagged EC12_WT_ was determined by coomassie blue staining and occasionally confirmed by western blot analysis with antibody specific for the 6xHis tag as described in “Materials and Methods”. The cellular lysates prepared from the EC12_WT_ expressing BL21 (DE3) pLysS bacteria were first purified on a HisTrap™ HP column using a AKTA pure chromatography system. Fractions contained 6xHis tagged EC12_WT_ were pooled and subsequently purified using a HiTrap™ Q HP, an anion exchange chromatographic column to improve the purity of EC12_WT_. Afterwards, 6xHis tagged EC12_WT_ containing fractions were pooled and dialyzed overnight in the presence of TEV protease to remove the 6xHis tag followed by the second HisTrap™ HP purification. The untagged EC12_WT_ was collected in flow-through sample and the TEV protease was eluted later with imidazole containing buffer. (B) A representative coomassie blue-stained SDS-PAGE. Image for protein samples collected after HisTrap chromatography following TEV protease (TEV) cleavage. Protein samples are indicated as Sample without TEV; In for input samples after TEV protease incubation; FT for flow-through sample; W for sample in wash; 20%B and 40%B for samples eluted with buffers containing 20% and 40% of buffer B which contains 1 M imidazole, respectively. (C) Activation of Cry1Ab toxin with the trypsin. Purification of trypsin-activated Cry1Ab toxin was performed according to the procedure described in “Materials and Methods”. Coomassie blue stained SDS-PAGE gel images show purified Cry1Ab (left panel) and EC12 _WT_ (right panel) with arrows indicating respective proteins. (D) Binding of purified EC12_WT_ to Cry1Ab in solution. Binding experiments were conducted by mixing various amounts of Cry1Ab with a fixed amount of EC12_WT_ at room temperature for 15 min, and *vice versa*. The reaction samples were resolved on a 6% native PAGE gel. Shown are the gel images after silver staining. E: EC12_WT_ only; C: Cry1Ab only; lanes 3 to 8: 41.1 μM of EC12 mixed with 1.25 μM, 2.5 μM, 3.7 μM, 4.9 μM, 6.2 μM and 9.3 μM of Cry1Ab, respectively; lanes 9 through 14: 12.4 μM of Cry1Ab mixed with 4.1 μM, 8.2 μM, 16.4 μM, 20.5 μM, 30.8 μM, and 41 μM of EC12_WT_, respectively. Arrows indicate the Cry1Ab, Cry1Ab/EC12_WT_ complex (Complex) and EC12_WT_ on the gel.

Previous studies have shown that Cry toxin binding is located in a finite region within EC12 of BT-R_1_, the cadherin GPCR for Cry1Ab [3–7,15]. However, none of these studies examined binding activity using purified toxin, EC12 and the toxin-EC12 complex in solution. In the present study, we investigated the direct binding of purified EC12_WT_ to Cry1Ab in solution. The procedure used to purify the EC12_WT_ peptide is outlined in Figure 1A and involved a combination of affinity (HisTrap), anion exchange (HiTrap Q) and affinity (HisTrap) chromatographic columns. Recombinant 6xHis-tagged EC12_WT_ contains a recognition sequence and specific cleavage site (ENLYFQ*GS, *cleavage site) for a TEV protease [16]. The predicted molecular weight of EC12_WT_ is 13.4-kDa. Results in Figure 1B show that a 13-kDa protein was generated by incubating with TEV protease and eluted in the flow-through sample (FT), suggesting that the 6xHis tag was removed from EC12_WT_. The proteins that eluted in buffer containing imidazole most likely are 6xHis-tagged TEV protease (20%B and 40%B). Trypsin activation and HiTrap Q anion exchange chromatographic purification of native Cry1Ab also was carried out using a procedure described in the “Materials and Methods”. Figure 1C reveals that Cry1Ab and EC12_WT_ were purified successfully. To test direct binding of Cry1Ab to EC12_WT_, the two proteins were mixed together in solution at different molar ratios and samples were separated on native PAGE gels. Figure 1D shows that both soluble EC12_WT_ (E) and Cry1Ab (C), indeed, were homogenous. Lanes 3 through 8 in Figure 1D display gradual formation of a band representing increasing amounts of Cry1Ab in the presence of a fixed amount of EC12_WT_. The band exhibits slightly faster mobility than Cry1Ab on the native gel. The reason for the observed shift in mobility of Cry1Ab relative to EC12 most likely is due to the pI of the two molecules, which, in a non-denaturing gel is reflective of both the mass and charge of the molecules. The pI for Cry1Ab is 6.5, for EC12 is 4.75 and for the Cry1Ab/EC12 complex is 5.75. Conversely, mixing increasing amounts of EC12_WT_ with a fixed amount of Cry1Ab produced a band with similar mobility (lanes 9 through 14). Both results signify formation of a Cry1Ab/EC12_WT_ complex. EC12_WT_ began to appear as faint band in lane 12 and increased in intensity in lanes 13 and 14, suggesting that binding to the Cry1Ab reached saturation.

**Figure 2:**
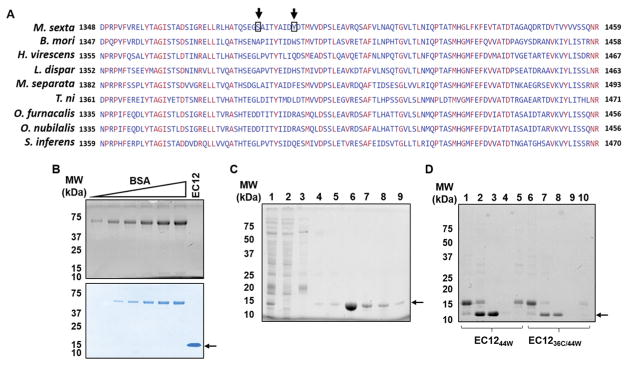
Comparison of wild-type and mutant EC12 peptides generated by site-directed mutagenesis. (A) Multiple sequence alignment of EC12 and corresponding regions in various moth cadherin GPCRs. Amino acid sequences for various moth cadherin GPCRs were aligned using Multiple Alignment tool developed by the National Center for Biotechnology Information of the United States of America (http://ncbi.nlm.nih.gov). Identical amino acid residues across all the species are indicated in red. (B) Detection of the EC12_WT_ protein using stain-free (top panel) and coomassie blue techniques (bottom panel). Purified EC12_WT_ was resolved on a SDS-PAGE gel along with increasing amounts of bovine serum albumin (BSA) as indicated on the image. (C) A representative stain-free image of a SDS-PAGE gel of EC12_36C/44W_ peptide samples is shown. Peptides were purified by HisTrap chromatography. Lane 1: input bacterial lysate; lane 2: flow-through; lane 3: column wash; lane 4 to 9: fractions eluted using an imidazole gradient of 2 mM to 1M. Arrow indicates EC12_36C/44W_. (D) A stain-free SDS-PAGE gel image showing both EC12_44W_ and EC12_36C/44W_ after the last HisTrap chromatographic procedure to remove the 6xHis tag and the 6xHis-tagged TEV protease. Lanes 1 to 5 contain protein samples for EC12_44W_ and lanes 6 to 10 contain proteins samples for EC12_36C/44W_ as indicated. Lanes 1 and 6: samples without TEV protease; lanes 2 and 7: input samples with TEV protease; lanes 3 and 8: flow-through samples; lanes 4 and 9: column wash samples; lanes 5 and 10: elution with buffer containing 0.2 M imidazole.

**Figure 3:**
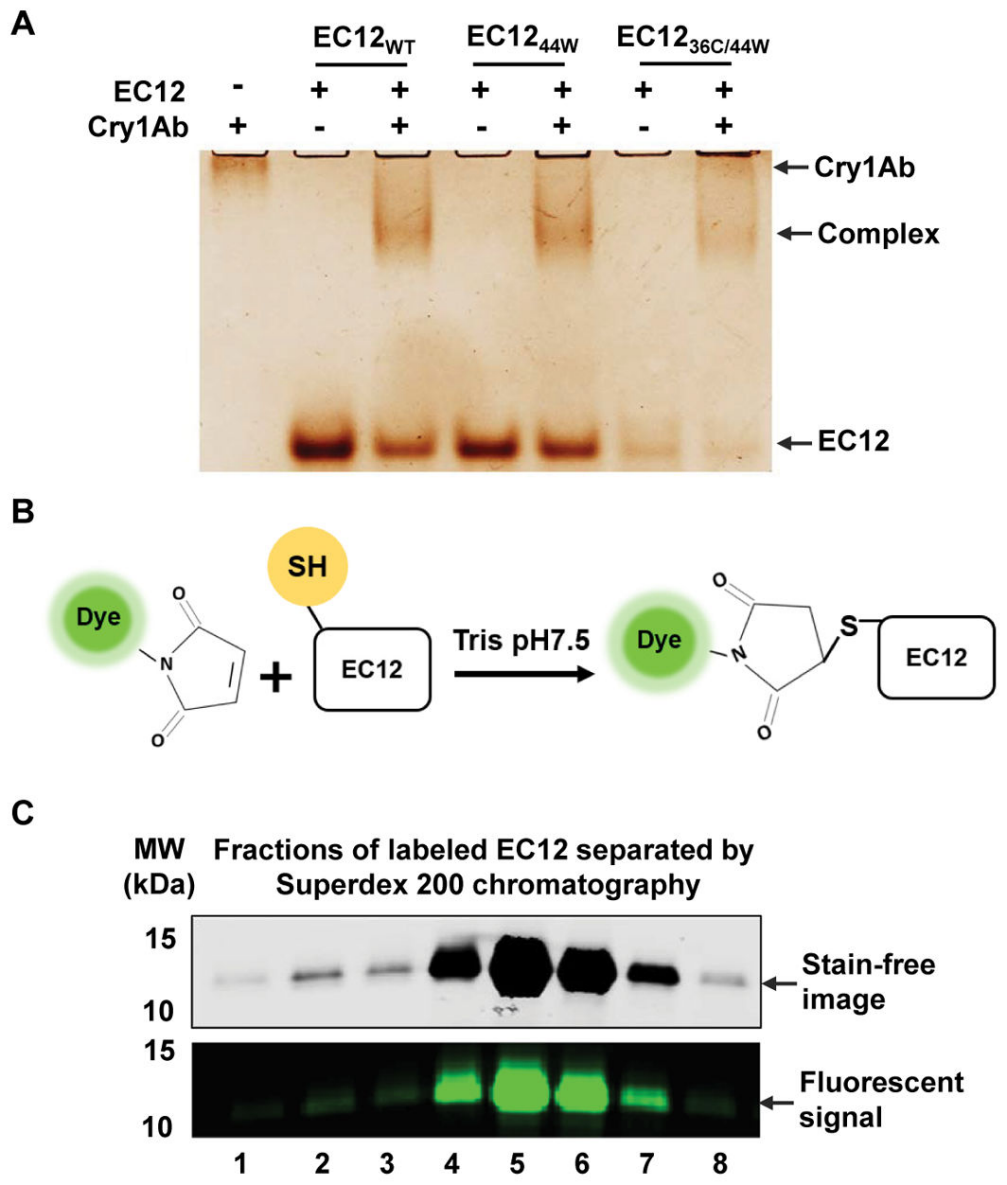
Fluorescent labeling of mutant EC12 peptide. (A) EC12_WT_ and mutant EC12 show similar binding capacity to Cry1Ab. Binding reactions of the EC12 peptides to Cry1Ab were conducted at room temperature for 15 min. Indicated samples were resolved on a native gel and silver stained afterwards. (B) Reaction showing the irreversible labeling of the cysteine residue on EC12_36C/44W_. “Dye” refers to Alexa-488, a fluorescent dye, which is conjugated to the maleimide moiety. Maleimide crosslinks the free thiol group depicted on the EC12 peptide. (C) Separation of Alexa-488 labeled EC12_36C/44W_ from free dye molecules by size exclusion chromatography. The peak protein fractions were resolved by SDS-PAGE. Stain-free images (top panel) were captured using a ChemiDoc MP Imaging system (Bio-Rad) and fluorescent images (bottom panel) recorded using a Typhoon FLA 9500 fluorescent scanner (GE Healthcare Life Science).

**Figure 4:**
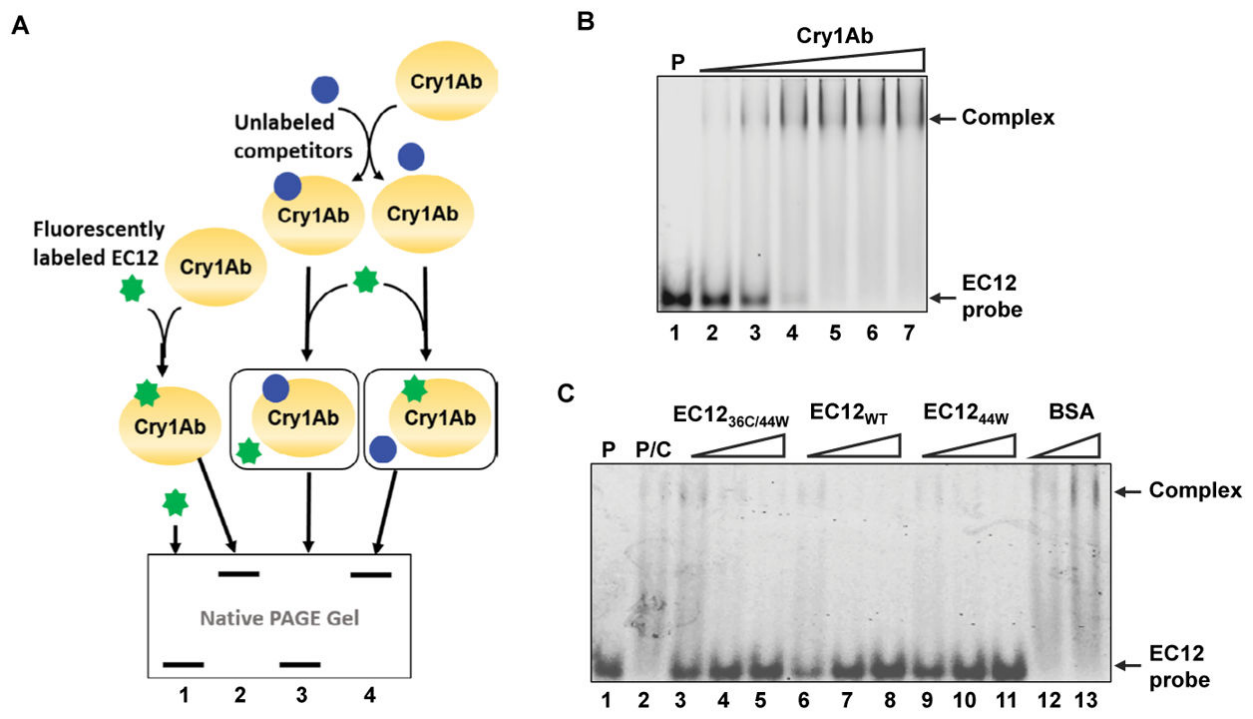
Detection of competitive binding with fluorescently labeled EC12. (A) Schematic of competitive binding reactions between EC12 and Cry1Ab. Labeled EC12_36C/44W_ (EC12 probe or probe) is depicted as a green “star” and, when not bound to Cry1Ab, appears as fluorescent bands at the bottom of a native gel (lanes 1 and 3). On the other hand, binding of the probe to Cry1Ab results in the formation of a protein complex shown as fluorescent bands at the top of a native gel (lanes 2 and 4). It is feasible that potential competitors (blue circles) could bind to Cry1Ab prior to the addition of the EC12 probe. As seen in Fig. 4A, two alternatives are possible. One alternative is that a competitor occupies the same binding site for the probe on the Cry1Ab toxin, preventing its binding to Cry1Ab (lane 3). The other alternative is that a protein may not be able to bind to Cry1Ab, thus allowing binding of the probe to the toxin (lane 4). (B) A representative fluorescent image demonstrating the binding of EC12 probe to Cry1Ab. Lane 1 (P): 2 μM EC12 probe; Lanes 2 to 7: 2 μM EC12 probe incubated with 1, 2, 3, 4, 5 and 6 μM Cry1Ab, respectively. (C) Competition binding experiments to determine the binding specificity of EC12 to Cry1Ab toxin. A representative fluorescent image of indicated protein samples on a native gel is shown. In these reactions, unlabeled EC12 molecules and bovine serum albumin (BSA) with various molar ratios to the EC12 probe was incubated with Cry1Ab (500 nM) at room temperature first. The EC12 probe was later added to the reaction. Reaction samples were resolved on a native gel and fluorescent image was scanned. Lane 1 (P): EC12 probe; lane 2 (P/C): EC12 probe mixed with Cry1Ab; lanes 3–11: Cry1Ab was incubated with 4-, 8- and 16- fold of unlabeled EC12_36C/44W_ (lanes 3–5), EC12_WT_ (lanes 6–8), EC12_44W_ (lanes 9–11) and BSA at 16- and 48-fold (lanes 12 and 13) followed by the addition of the EC12 probe. Arrows indicate unbound EC12 probe and Cry1Ab/EC12 complex.

EC12_WT_ and Cry1Ab formed a stable complex in solution. In-solution binding assays utilizing purified proteins such as those described herein provide a means to investigate the nature of the Cry1Ab-EC12_WT_ interaction. More importantly, formation of a stable, soluble complex between Cry1Ab and EC12_WT_ will allow direct structure-function analysis of this interaction. The binding affinity of Cry1Ab toxin for full-length BT-R_1_ is quite high (1.0 nM as measured with radioactively labeled toxin) measured by using ^125^I-labeled Cry1Ab in an indirect detection assay [3]. Alternatively, maleimide-based conjugation of a fluorescent dye to cysteine residue(s) in target proteins affords an excellent way to measure protein-protein interaction without the use of radioisotopes or toxic chemicals [17]. However, EC12_WT_ does not contain a cysteine residue as shown by its sequence in Figure 2A. Based on multiple sequence alignment for EC12_WT_ and corresponding conserved regions among different moth species (Figure 2A), residues 36 and 44 were chosen for site-directed mutagenesis, i.e., serine was changed to cysteine and tyrosine was changed to tryptophan, respectively. Luckily, neither of these two residues was located within the N- and C-termini required for Cry1Ab binding. As seen in Figure 2B, EC12_WT_ was not visible by stain-free imaging although the peptide was detectable with coomassie blue and silver staining. As is widely recognized, both staining techniques are time-consuming and require toxic chemicals. Furthermore, quantification is complicated if a peptide such as EC12_WT_ does not contain a tryptophan residue. The stain-free imaging protocol described in Materials and Methods is based on tryptophan being present in the peptide. Changing the 44^th^ residue in EC12 from tyrosine to tryptophan (EC12_44W_), combined with the change of the 36^th^ residue from serine to cysteine (EC12_36C/44w_), facilitated peptide detection and quantification of the two peptides using the stain-free protocol (Figures 2C and D). Fortuitously, mutating these two residues did not affect Cry1Ab binding. EC12_44W_, EC12_WT_ and EC12_36C/44W_ all appeared to be homogenous as reflected by silver staining in a native gel, and each peptide bound Cry1Ab to form a complex in a similar manner (Figure 3A). Subsequently, purified EC12_36C/44W_ was labeled with Alexa-488 in a maleimide-mediated conjugation reaction (Figure 3B). The resulting EC12 probe was purified on a size-exclusion chromatographic column and shown to exhibit a strong fluorescent signal (Figure 3C), enabling highly sensitive detection of EC12 and binding of the peptide to Cry1Ab toxin. As is true for most binding assays, there are alternative outcomes that should be considered. Figure 4A presents four possible results for Cry1Ab binding using the EC12 probe. The first alternative is that the probe does not bind and is detectable at the bottom of the gel (lane 1). Alternative two would involve binding of the probe to Cry1Ab producing a Cry1Ab/EC12 complex detectable as a fluorescent band at the top of the gel (lane 2). If the probe fails to bind because an unlabeled protein competes with the probe, the competitor could occupy the probe-binding site on Cry1Ab. The result would be migration of the probe to the bottom of the gel as seen in lane 3. The fourth alternative is that a potential competitor protein may not bind to Cry1Ab, thus allowing the probe to bind the toxin (see band at the top of the gel in lane 4). To test these potential outcomes, several binding experiments were conducted using the fluorescent EC12 probe in the presence of increased amounts of the toxin. Figure 4B illustrates that the fluorescent band representing the probe at the bottom of the native gel (lane 1) gradually disappeared with increasing amounts of Cry1Ab in the reaction (lanes 2–7) with a concomitant increase in Cry1Ab/EC12 complex formation as indicated by appearance of a band near the top of the gel. Because these two alterations occurred simultaneously, we believe that the changes in fluorescence implicate direct probe binding to the toxin. Obviously, fidelity of the fluorescent probe is an index of its utility in specifying Cry1Ab binding to EC12 as well as to other related or unknown proteins. The binding affinity of Cry1Ab to EC12 was challenged using unlabeled EC12 peptides mixed with the fluorescent EC12 probe. As expected, the EC12 probe disappeared in the presence of Cry1Ab (500 nM; Figure 4C, lane 2) with the appearance of a slow-mobility band similar to the complex band in Figure 4B, indicating formation of a Cry1Ab/EC12 complex. Unsurprisingly, the EC12 probe persisted in the presence of increasing amounts (4-, 8- and 16-fold) of unlabeled EC12_36C/44W_ (lanes 3–5), EC12_WT_ (lanes 6–8) and EC12_44W_ (lanes 9–11). On the other hand, intensity of the complex decreased with the addition of EC12 specific peptides. The response was dose-dependent. Clearly, the various EC12 peptides have similar binding affinity for the Cry1Ab toxin. Bovine serum album (BSA), a totally unrelated protein, was not competitive (lanes 12 and 13). We believe that EC12 binding is sequence dependent and highly specific as previously suggested [8]. The labeled EC12_36C/44W_, as constructed, is a valuable probe for assessing specificity, affinity and the kinetics of binding.

## Discussion

Various species of Bt have been used commercially to control insects that pose harm to agricultural crops throughout the world. Insecticidal activity is based on the ability of specific Cry toxins to selectively kill insects. Some toxins are specific to moths whereas others are specific to beetles and mosquitoes. The Cry1Ab toxin of *Bacillus thuringiensis* subsp. *berliner* exerts its insecticidal activity by binding to the cadherin GPCR BT-R_1_ localized in the midgut epithelial cells of the moth *Manduca sexta*. This interaction happens with very high affinity (Kd~ 1 nM) [4]. Consequently, the bound BT-R_1_ initiates an intracellular signaling cascade to bring about killing of the epithelial cells and the whole insect. Elucidating the underlying amino acid sequences involved in binding of the Cry1Ab should provide important information about the determinants of binding specificity and the molecular mechanism of the interaction. Numerous studies have been conducted to determine the specific toxin-binding region in BT-R_1_ [4,8,12,15,18]. Two fairly recent studies have demonstrated that the toxin-binding region is localized within the EC12 module of BT-R_1_ [8,15]. This region also conveys Cry1Ab toxicity. Based on the findings from previous cytological and biochemical investigations, we conducted experiments to assess direct interaction between the EC12 of BT-R_1_ with Cry1Ab. Using highly purified EC12 and Cry1Ab proteins, we demonstrated that these two proteins, indeed, formed a complex. In contrast to approaches used in many previous studies, we visualized formation of the complex directly on native PAGE gels, demonstrating that the complex is soluble and stable. This result provides a basis for future studies regarding the structural features of the Cry1Ab-EC12 interaction.

To improve sensitivity of detection, we engineered mutations on two amino acid residues (36^th^ and 44^th^ residues) in EC12. The resulting EC12_44W_ and EC12_36C/44W_ peptides were detectable by stain-free imaging, suggesting that these residues were present on the surface of EC12. Mutagenesis of the two amino acid residues did not interfere with binding of the Cry1Ab toxin (Figures 3A and 4C), indicating that the toxin-binding region is not close to these two residues. Cysteine-based fluorescent labeling of protein has been widely used in various studies of protein-protein and protein-DNA/RNA interactions because of the high sensitivity and low toxicity of various fluorophores [19]. Therefore, we tested binding of Cry1Ab with labeled EC12 probe by using an in-solution HI-FI assay system [20]. Because the labeled cysteine was not located in the Cry1Ab binding region, there was no measurable change in fluorescent intensity in those wells containing increasing amounts of Cry1Ab toxin (results not shown). However, resolving the samples on a native gel rendered images of Cry1Ab-EC12 complex formation with simultaneous disappearance of the probe as seen in Figure 4B. Obviously, location of the labeled residue dictates the method of detection. By using the EC12 probe, we will be able to directly and accurately quantify binding reactions between the Cry1Ab toxin and the EC12 probe. Furthermore, as demonstrated in Figure 4C, we will be able to perform large-scale screening of ligand peptides for their capacity to bind the EC12 probe. In summary, we have established an in-solution binding assay using purified Cry1Ab and EC12 proteins. Formation of the stable Cry1Ab/EC12 complex most assuredly will expedite future structural analysis of the complex. Importantly, the EC12 mutants generated by site-directed mutagenesis exhibited Cry1Ab binding capacity similar to the wild-type protein (EC12_WT_). The tyrosine to tryptophan change greatly improves protein traceability throughout the process. Maleimide-mediated fluorescent labeling of the solitary cysteine residue of EC12_36C/44W_ enabled competitive binding assays for determining binding specificity. We believe that this strategy can be used to develop platforms for high throughput screening of various ligand peptides.

## References

[1] Ibrahim MA, Griko N, Junker M, Bulla LA (2010). Bacillus thuringiensis: a genomics and proteomics perspective. Bioeng Bugs.

[2] Vadlamudi RK, Ji TH, Bulla LA (1993). A specific binding protein from Manduca sexta for the insecticidal toxin of Bacillus thuringiensis subsp. berliner. J Biol Chem.

[3] Vadlamudi RK, Weber E, Ji I, Ji TH, Bulla LA (1995). Cloning and expression of a receptor for an insecticidal toxin of Bacillus thuringiensis. J Biol Chem.

[4] Dorsch JA, Candas M, Griko NB, Maaty WSA, Midboe EG (2002). Cry1A toxins of Bacillus thuringiensis bind specifically to a region adjacent to the membrane-proximal extracellular domain of BT-R(1) in Manduca sexta: involvement of a cadherin in the entomopathogenicity of Bacillus thuringiensis. Insect Biochem Mol Biol.

[5] Gahan LJ, Gould F, Heckel DG (2001). Identification of a gene associated with Bt resistance in Heliothis virescens. Science.

[6] Jurat-Fuentes JL, Gahan LJ, Gould FL, Heckel DG, Adang MJ (2004). The HevCaLP protein mediates binding specificity of the Cry1A class of Bacillus thuringiensis toxins in Heliothis virescens. Biochemistry.

[7] Nagamatsu Y, Toda S, Koike T, Miyoshi Y, Shigematsu S (1998). Cloning, sequencing, and expression of the Bombyx mori receptor for Bacillus thuringiensis insecticidal CryIA(a) toxin. Biosci Biotechnol Biochem.

[8] Griko NB, Young LR, Zhang X, Carpenter L, Candas M (2007). Univalent binding of the Cry1Ab toxin of Bacillus thuringiensis to a conserved structural motif in the cadherin receptor BT-R_1_. Biochemistry.

[9] Zhang X, Candas M, Griko NB, Taussig R, Bulla LA (2006). A mechanism of cell death involving an adenylyl cyclase/PKA signaling pathway is induced by the Cry1Ab toxin of Bacillus thuringiensis. Proc Natl Acad Sci U S A.

[10] Zhang X, Griko NB, Corona SK, Bulla LA (2008). Enhanced exocytosis of the receptor BT-R(1) induced by the Cry1Ab toxin of Bacillus thuringiensis directly correlates to the execution of cell death. Comp Biochem Physiol B Biochem Mol Biol.

[11] Griko N, Candas M, Zhang X, Junker M, Bulla LA (2004). Selective antagonism to the cadherin BT-R_1_ interferes with calcium-induced adhesion of epithelial membrane vesicles. Biochemistry.

[12] Zhang X, Candas M, Griko NB, Young LR, Bulla LA (2005). Cytotoxicity of Bacillus thuringiensis Cry1Ab toxin depends on specific binding of the toxin to the cadherin receptor BT-R_1_ expressed in insect cells. Cell Death Differ.

[13] Peranen J, Rikkonen M, Hyvönen M, Kääriäinen L (1996). T7 vectors with modified T7lac promoter for expression of proteins in Escherichia coli. Anal Biochem.

[14] Ladner CL, Yang J, Turner RJ, Edwards RA (2004). Visible fluorescent detection of proteins in polyacrylamide gels without staining. Anal Biochem.

[15] Hua G, Jurat-Fuentes JL, Adang MJ (2004). Bt-R_1_a extracellular cadherin repeat 12 mediates Bacillus thuringiensis Cry1Ab binding and cytotoxicity. J Biol Chem.

[16] Phan J, Zdanov A, Evdokimov AG, Tropea JE, Peters HK (2002). Structural basis for the substrate specificity of tobacco etch virus protease. J Biol Chem.

[17] Kim Y, Ho SO, Gassman NR, Korlann Y, Landorf EV (2008). Efficient site-specific labeling of proteins via cysteines. Bioconjug Chem.

[18] Gomez I, Dean DH, Bravo A, Soberón M (2003). Molecular basis for Bacillus thuringiensis Cry1Ab toxin specificity: two structural determinants in the Manduca sexta Bt-R_1_ receptor interact with loops alpha-8 and 2 in domain II of Cy1Ab toxin. Biochemistry.

[19] Kuan SL, Wang T, Weil T (2016). Site-Selective Disulfide Modification of Proteins: Expanding Diversity beyond the Proteome. Chemistry.

[20] Winkler DD, Luger K, Hieb AR (2012). Quantifying chromatin-associated interactions: the HI-FI system. Methods Enzymol.

